# Protective Effects of Dihydrocaffeic Acid, a Coffee Component Metabolite, on a Focal Cerebral Ischemia Rat Model

**DOI:** 10.3390/molecules200711930

**Published:** 2015-06-30

**Authors:** Kyungjin Lee, Beom-Joon Lee, Youngmin Bu

**Affiliations:** College of Korean Medicine, Kyung Hee University, 26 Kyungheedae-ro, Dongdaemun-gu, Seoul 130-701, Korea; E-Mails: niceday@khu.ac.kr (K.L.); franchisjun@naver.com (B.-J.L.)

**Keywords:** dihydrocaffeic acid, cerebral ischemia, brain edema, blood brain barrier, matrix metalloproteinase

## Abstract

We recently reported the protective effects of chlorogenic acid (CGA) in a transient middle cerebral artery occlusion (tMCAo) rat model. The current study further investigated the protective effects of the metabolites of CGA and dihydrocaffeic acid (DHCA) was selected for further study after screening using the same tMCAo rat model. In the current study, tMCAo rats (2 h of MCAo followed by 22 h of reperfusion) were injected with various doses of DHCA at 0 and 2 h after onset of ischemia. We assessed brain damage, functional deficits, brain edema, and blood-brain barrier damage at 24 h after ischemia. For investigating the mechanism, *in vitro* zymography and western blotting analysis were performed to determine the expression and activation of matrix metalloproteinase (MMP)-2 and -9. DHCA (3, 10, and 30 mg/kg, i.p.) dose-dependently reduced brain infarct volume, behavioral deficits, brain water content, and Evans Blue (EB) leakage. DHCA inhibited expression and activation of MMP-2 and MMP-9. Therefore, DHCA might be one of the important metabolites of CGA and of natural products, including coffee, with protective effects on ischemia-induced neuronal damage and brain edema.

## 1. Introduction

Coffee is a widely consumed beverage worldwide. The beneficial activities on human diseases, especially on vascular diseases and cerebral ischemia, have been well documented [1]. The major component is thought to be caffeine, but chlorogenic acid (CGA), which is present in higher amounts than caffeine, is reported to also be a major effective compound in coffee [1,2]. The pharmacological activities of CGA have been studied extensively [1,3]. We previously reported the protective effects of CGA against brain edema, as well as brain infarction, in a transient focal cerebral ischemia rat model via inhibition of matrix metalloproteinase (MMP)-2 and -9 expression and activities [4]. Furthermore, it is well recognized that CGA is broken down to many metabolites after administration to animals and humans [5,6,7]. To expand upon the results of our previous report, we preliminarily screened the effects of several CGA metabolites and found that caffeic acid (CA) and dihydrocaffeic acid (DHCA) showed potent protective effects (data not shown). CA is a well-known anti-oxidant component and has protective effects on various cerebral ischemia animal models [8,9]. Moreover, caffeic acid phenethyl ester, which is the mostly studied derivative of CA, showed protective effects on various cerebral ischemia models through anti-inflammatory and anti-oxidative mechanisms [8,9]. DHCA, a metabolite of CGA (also of CA), is also a well-known anti-oxidant component, and there are several studies on the beneficial activities of DHCA, such as anti-oxidative [10,11], anti-Alzheimer’s [12], neuroprotective [13], arousal [14], and lipid-lowering effects [15].

However, to our knowledge, the effect of DHCA on cerebral infarction and brain edema in a cerebral ischemia animal model has not been reported yet. Therefore, we evaluated the effects of DHCA on brain damage, behavioral deficits, brain water content (BWC), and Evans blue (EB) extravasation in the same model used in our previous study. Further, we analyzed the effects of DHCA on MMP-2 and MMP-9 expression and activity to study the mechanisms of action [4].

## 2. Results and Discussion

### 2.1. Results

#### 2.1.1. Effect of DHCA on Brain Infarct Volume and Sensory-Motor Function Deficit

**Figure 1:**
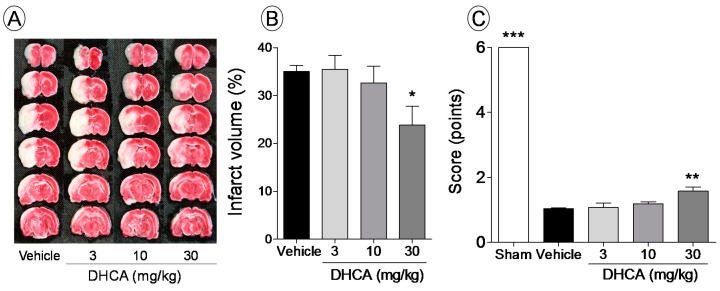
The protective effect of dihydrocaffeic acid (DHCA) on cerebral infarct volume (**A**,**B**) and sensory-motor function deficits in a middle cerebral artery occlusion (MCAo) rat model (**C**). Images represent 2,3,5-triphenyltetrazolium chloride (TTC)-stained brain sections from vehicle- and DHCA-treated (3, 10, and 30 mg/kg, i.p.) groups (**A**). Graphs show the percentage of brain infarct volume (**B**) and the behavioral score for each group (**C**). Sham indicates sham-operated group. Data represent the mean ± SEM (*n* = 7–10). * *p* < 0.05, ** *p* < 0.01, and *** *p* < 0.001 compared with the vehicle-treated group.

In the brain of the vehicle-treated rat, white area (infarcted area) could be seen in whole sections, which extended from the ischemic core to the penumbra, after middle cerebral artery occlusion (MCAo). However, DHCA-treated rats showed less brain damage than vehicle-treated rats (Figure 1A). DHCA (3, 10, and 30 mg/kg, i.p.) showed dose-dependent effects on brain infract volume (35.4% ± 3.1%, 32.6% ± 3.1%, and 23.8% ± 4.1% of infarct volume, respectively), compared to the vehicle (35.0% ± 1.3% of infarct volume), and the 30 mg/kg DHCA-treated group showed a 31.9% protective effect compared to the vehicle-treated group (*p* < 0.05; Figure 1B). Significantly lower scores in the balance-beam test were observed in the vehicle-treated group compared to the sham group (1.0 ± 0.1 *vs.* 6.0 ± 0.0 points, *p* < 0.01). While, DHCA treatment at 3, 10, and 30 mg/kg showed dose-dependent improvement in behavioral score (1.1% ± 0.1%, 1.2% ± 0.0%, and 1.6% ± 0.1%, respectively; *p* < 0.05 in the 30 mg/kg DHCA-treated group; Figure 1C).

#### 2.1.2. Effect of DHCA on BWC and EB Extravasation

**Figure 2:**
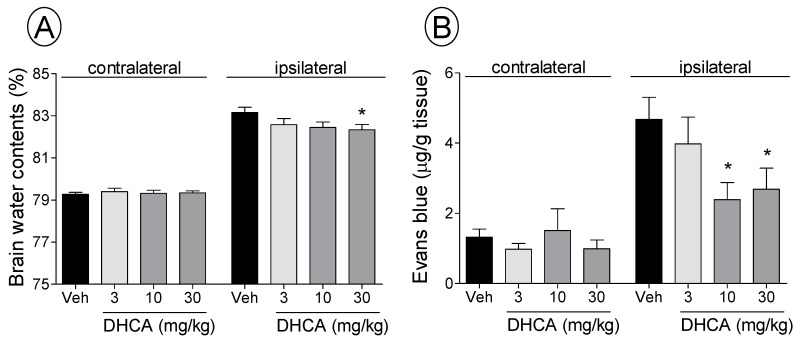
The effect of dihydrocaffeic acid (DHCA) on brain water content (BWC) (**A**) and Evans Blue (EB) leakage after middle cerebral artery occlusion (MCAo) (**B**). Veh is vehicle-treated group. Data represent the mean ± SEM (*n* = 8). * *p* < 0.05 compared with the contralateral hemisphere of the vehicle-treated group.

Brain water contents at 22 h after ischemia in the ipsilateral hemisphere were more than in the contralateral hemisphere. All doses of DHCA decreased mean BWC in the ipsilateral hemisphere (vehicle: 83.2% ± 0.2%, 3 mg/kg DHCA: 82.6% ± 0.3%, 10 mg/kg DHCA: 82.4% ± 0.3%, 30 mg/kg DHCA: 82.3% ± 0.2%; *p* < 0.05 in all DHCA-treated groups; Figure 2A). There were no significant differences in BWC in the contralateral hemisphere between groups.

The MCAo rat model also showed greater EB extravasation in the ipsilateral hemisphere than in the contralateral hemisphere. The vehicle-treated group showed EB content f 4.6 ± 0.7 µg/g tissue in the ipsilateral hemisphere, whereas the 3, 10, and 30 mg/kg DHCA-treated groups showed a dose-dependent decrease (3.9 ± 0.8, 2.4 ± 0.5, and 2.7 ± 0.5 µg/g tissue, respectively; *p* < 0.05 in the 10 and 30 mg/kg DHCA-treated group; Figure 2B). There were no significant differences in EB content in the contralateral hemisphere between groups.

#### 2.1.3. Effect of DHCA on MMP-2 and MMP-9 Protein Expression

**Figure 3:**
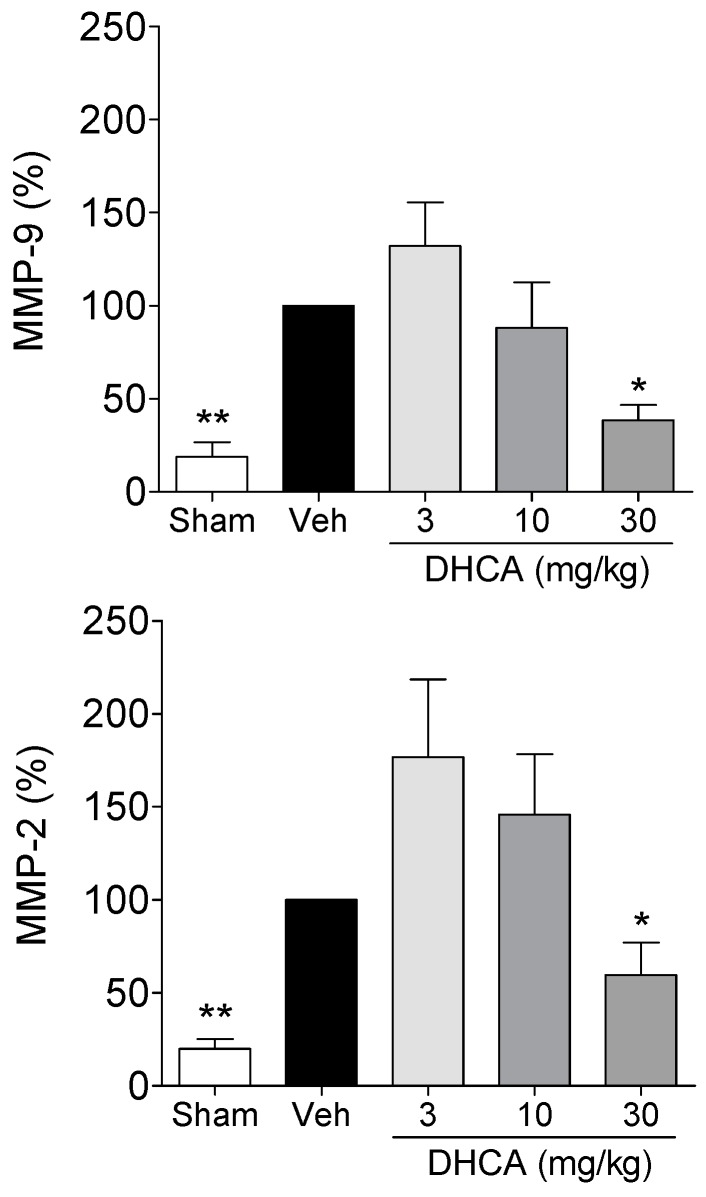
The effect of dihydrocaffeic acid (DHCA) on matrix metalloproteinase (MMP)-2 and MMP-9 protein expression at 24 h after middle cerebral artery occlusion (MCAo). Veh is vehicle-treated group. Data represent the mean ± SEM (*n* = 6 per group) and are normalized as a percentage of the vehicle-treated group. * *p* < 0.05 and ** *p* < 0.01 compared with the vehicle-treated group.

MMP-2 and MMP-9 protein levels in the vehicle-treated group at 24 h after ischemia were upregulated compared to that of the sham-operated group. Treatment with 3, 10, and 30 mg/kg DHCA dose-dependently downregulated MMP-2 and MMP-9 protein expressions (*p* < 0.05, vehicle *vs.* 30 mg/kg DHCA-treated group; Figure 3).

#### 2.1.4. Effect of DHCA on MMP-2 and MMP-9 Activity

**Figure 4:**
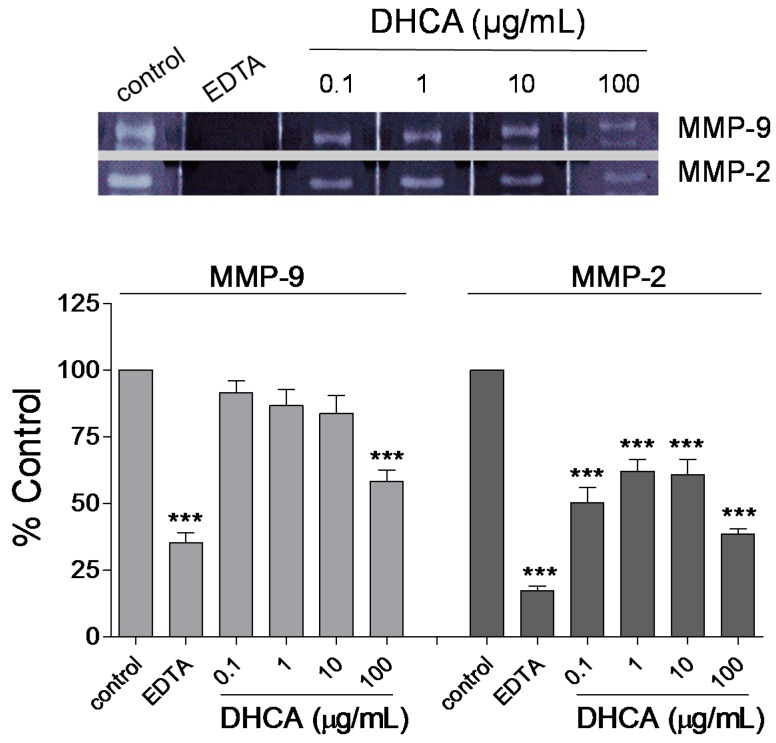
The effect of dihydrocaffeic acid (DHCA) on matrix metalloproteinase (MMP)-2 and MMP-9 activity assessed by *in vitro* zymography. The image is representative of a typical zymogram for MMP-2 and MMP-9. The graphs show the effect of DHCA on MMP-2 and MMP-9 activity. EDTA is a positive control (20 mM). Data are represented as mean ± SEM (*n* = 3 per group) and are normalized as a percentage of the vehicle-treated group. *** *p* < 0.001 compared with the vehicle-treated group.

DHCA concentration-dependently inhibited MMP-2 and MMP-9 activities as assessed by *in vitro* zymography. DHCA (100 µg/mL) significantly inhibited MMP-2 and MMP-9 activities. The positive control (20 mM EDTA) almost completely inhibited the activity of MMP-2 and MMP-9 (Figure 4).

### 2.2. Discussion

In the present study, DHCA inhibited brain infarct volume, sensory-motor function deficit, brain edema, and BBB damage via the inhibition of MMP-2 and -9 expressions and activities in a MCAo rat model. Intraperitoneal injection of DHCA (3, 10, and 30 mg/kg) dose-dependently protected against brain damage and improved the functional behavior of rats. The current animal model is well-known and extensively studied in the field of brain stroke [16,17], which is characterized by the type of cell death, including necrotic type in the ischemic core and apoptotic type in the penumbra [18]. The former is known to be recovered by recanalization within 3 h of onset, and the latter could be recovered after 3 h of onset [19]. DHCA did not reduce the damage in the ischemic core but reduced that in the penumbra. The current results were supported by results of the balance-beam test. Although we did not compare the activities of CGA and DHCA directly, CGA might show more activity than DHCA [4], which suggests that the effects of CGA partly come from DHCA as well as other metabolites.

In the current study, DHCA dose-dependently reduced BWC and decreased EB extravasation in the ipsilateral hemisphere at 24 h after MCAo, which were measured to investigate the effects on brain edema and the BBB damage, respectively [20,21]. The pathophysiological characteristics of brain edema and BBB damage have been well investigated [22], and reducing brain edema and BBB damage could be indirect mechanisms of the effects of DHCA on stroke [4,23,24]. The current results suggested that the inhibitory effects of DHCA on brain edema might originate from its ability reduce breakdown of the BBB.

DHCA dose-dependently downregulated MMP-2 and MMP-9 expression compared to the vehicle-treated group. DHCA inhibited MMP-2 and MMP-9 activity in *in vitro* zymography assays. MMP-2 and MMP-9 are reported to be the main candidates for the mechanisms of BBB breakdown [21,25,26,27,28] and are known to directly degrade the extracellular matrix and lead to BBB damage followed by brain edema. Inhibition of MMP-2 and MMP-9 could reduce brain edema via inhibiting BBB damage in the acute phase of brain ischemia [20,21,25,29]. Brain edema is reported to aggravate brain damage by reduction of blood flow to the damaged area due to increase of cranial pressure [20,21,22]. The current results are supported by radical scavenging activities of DHCA because, free radical is reported to be a major mediator in MMPs upregulation [30,31], and by the direct MMPs inhibitory activities of CGA and CA, which are the precursors of DHCA [4,32,33,34].

## 3. Experimental Section

### 3.1. DHCA Preparation

DHCA (purity > 98%) was purchased from Sigma-Aldrich (St. Louis, MO, USA). DHCA was dissolved in water and filtered for preparation of an injectable solution.

### 3.2. Animals

Sprague-Dawley rats (SD rat, 8-weeks-old, Samtaco, Osan, Gyeonggi Province, Korea) were acclimatized for 1 week under conditions of controlled temperature (22 ± 2 °C), constant humidity, and a 12-h light/dark cycle, and food and water were made available *ad libitum*. Rats weighing 290–310 g were used after acclimatization for the study. All surgical procedures were conducted according to the animal welfare guidelines of the NIH; this study was approved by the Kyung Hee University Institutional Animal Care and Use Committee (KHUASP(SE)-12-027).

### 3.3. Focal Cerebral Ischemia Rat Model Induction and Treatment

Focal cerebral ischemia was induced by MCAo using the intraluminal suture method, which was described previously [4,16]. Briefly, rats were anesthetized with isoflurane in N_2_O and O_2_ (70:30). MCAo was induced by inserting sutures (360 µm in thicknesses; Doccol Co., Sharon, NM, USA) 19–20 mm from the bifurcation of the internal and external carotid arteries for 2 h. Reperfusion was achieved by retracting the sutures. In the sham-operated group, the procedure was the same except for the probe insertion length (10 mm). The vehicle-treated group was injected intraperitoneally with water (3 mL/kg) twice at 0 h and 2 h after ischemia. DHCA-treated groups were treated various dosages of DHCA (3, 10, and 30 mg/kg) using the same method as the vehicle-treated group. The rectal temperatures of rats were controlled throughout surgery and for 6 h after surgery (37 ± 0.5 °C).

Rats were divided into four groups (vehicle-treated, 3, 10, and 30 mg/kg DHCA-treated) for measurement of infarct volume (*n* = 10), EB leakage (*n* = 8), and BWC (*n* = 8). Rats were divided into five groups (sham, vehicle-treated, 3, 10, and 30 mg/kg DHCA-treated) for assessment of sensory-motor function (*n* = 6 for sham, *n* = 10 for other groups) and western blotting for MMP-2 and MMP-9 (*n* = 6).

### 3.4. Balance Beam Test

The balance-beam test was performed at 22 h after ischemia, using a modified method of the previous method [4]. Briefly, rats were placed on a wooden square bar (2.5 cm width, 122 cm length, 42 cm height) and scored in a blinded fashion as follows: rats unable to stay on the beam for 30 s, 0; rats able to stay on the beam for 30 s, 1; rats able to turn to the left or right side of the beam and stay without walking, 2; rats able to turn left or right and walk on the beam with more than one step, 3; rats able to traverse the beam with more than 50% of foot slip of the affected hind limb, 4; rats able to transverse the beam with less than 50% of foot slip of the hind limb, 5; and rats able to traverse the beam with not more than one foot slip, 6.

### 3.5. Measurement of Infarct Volume

Rats were over-anesthetized with urethane (i.p., 1.25 g/kg) at 24 h after ischemia, and the brains were isolated quickly and cut into six coronal sections with 2-mm thickness, starting 4 mm from the rostral extremity of the frontal cortex. Sections were incubated with 2% 2,3,5-triphenyltetrazolium chloride (TTC; Sigma-Aldrich) in saline for 30 min at 37 °C. TTC-stained sections were imaged and analyzed using an image analysis system (Optimas; Media Cybernetics, Silver Springs, MD, USA). Correlated infarct volume (%) was calculated based on a reported method [35].

### 3.6. Measurement of BWC

The BWC was measured using a method previously described [4]. Briefly, rat brains were isolated quickly and divided into the ipsilateral and contralateral hemispheres at 24 h after ischemia. The wet weight of each hemisphere was measured within 90 s of isolation, and the dry weight was measured after drying the brain in an oven at 105 °C for 24 h. The BWC of each hemisphere was calculated as ([wet weight − dry weight]/wet weight] × 100%.

### 3.7. Measurement of EB Extravasation

EB extravasation was analyzed 24 h after MCAo using the method reported previously [10], with some modifications. EB dye (2%, 6 mL/kg body weight; Sigma-Aldrich) was injected slowly into the femoral artery at 21 h after occlusion and allowed to circulate for 3 h. After brain isolation, the pons and olfactory bulb were removed, and the brain was separated immediately into the ipsilateral and contralateral hemispheres. Each hemisphere was weighed, formamide was added (800 μL, Sigma-Aldrich), and then the hemisphere was homogenized. Each homogenized hemisphere was incubated at 60 °C for 24 h and then centrifuged at 14,000 rpm for 30 min. The absorption of the supernatant was measured at 610 nm with a spectrophotometer. A standard curve of EB in blank formamide was used to convert the absorbance into the concentration of EB dye. Data are presented as µg of EB dye per gram of tissue [10].

### 3.8. Western Blot

Brain tissues were isolated at 24 h after ischemia and lysed in triple-detergent lysis buffer (50 mM Tris-HCl, 150 mM NaCl, 0.1% sodium dodecyl sulfate (SDS), 1% NP-40; 0.02% sodium azide, 0.5% sodium deoxycholate, and 1 mM phenylmethylsulfonylfluoride; pH 8.0). Protein (80 μg for each sample) was separated using 12% SDS-polyacrylamide gel electrophoresis (SDS-PAGE) and transferred to a nitrocellulose membrane (Whatman, Dassel, Germany). The membrane was blocked with 5% skim milk and incubated with anti-rabbit primary antibodies against MMP-2 (1:1000; ABclonal Biotech Co., Ltd., Cambridge, MA, USA), MMP-9 (1:1000; Epitomics, Burlingame, CA, USA), or β-actin (1:2000; Cell Signaling, Beverly, MA, USA). Horseradish peroxidase-linked secondary antibody (1:2000; Cell Signaling, Beverly, MA, USA) was used. Proteins were detected by incubating blots with Rapid Step™ ECL Reagent (Calbiochem^®^, San Diego, CA, USA). Bands were analyzed using Quantity One 1-D analysis software (Bio-Rad, Hercules, CA, USA).

### 3.9. In Vitro Zymography

MMP-2 and MMP-9 activities were determined by gelatin zymographic analysis as described previously [4,36], with some modifications. Briefly, human recombinant MMP-2 and MMP-9 (6 ng and 0.6 ng per lane, respectively; Sigma-Aldrich) were isolated on an 8% SDS-PAGE gel (1-mm thickness) containing gelatin (2%). The gels were washed with 2.5% Triton X-100 and then incubated with the incubation buffer (0.05 M Tris-HCl (Invitrogen, San Diego, CA, USA) containing 0.005 M CaCl_2_, 0.2 M NaCl, (Merck, Darmstadt, Germany), and 0.02% NaN_3_; pH 7.5) at 37 °C for 20 h with or without DHCA (0.1, 1, 10, and 100 µg/mL) or EDTA (50 µM). The gels were stained with 0.05% Coomassie brilliant blue R-250 in 25% methanol and 10% acetic acid for 2 h at room temperature on a shaking platform and then destained with 4% methanol and 8% acetic acid for 30 min. The zymograms were scanned using the PowerLook 1000 scanner (UMAX, Dallas, TX, USA), and the gelatinolytic bands were analyzed using Quantity One 1-D analysis software (Bio-Rad).

### 3.10. Statistical Analysis

All results are presented as mean ± SEM, and comparisons between experimental groups were analyzed using one-way ANOVA followed by Dunnett’s test. Values of *p* < 0.05 were considered statistically significant.

## 4. Conclusion

Taken together, the current results suggested that the inhibitory effects of DHCA on BBB damage might result from a reduction in the production and activity of MMP-2 and MMP-9. Taken together, DHCA may be one of the major active metabolites and compounds of natural products, including coffee, responsible for the protective effects of CGA on ischemic stroke.
